# Flu Vaccination Attitudes, Behaviours, and Knowledge among Health Workers

**DOI:** 10.3390/ijerph17093185

**Published:** 2020-05-03

**Authors:** Antonella Arghittu, Marco Dettori, Antonio Azara, Davide Gentili, Antonello Serra, Bruno Contu, Paolo Castiglia

**Affiliations:** 1 Department of Biomedical Sciences, University of Sassari, 07100 Sassari, Italy; arghittu.antonella@gmail.com; 2 University Hospital in Sassari, 07100 Sassari, Italy; azara@uniss.it (A.A.); antonello.serra@aousassari.it (A.S.); bruno.contu@aousassari.it (B.C.); 3 Department of Medical, Surgical and Experimental Sciences, University of Sassari, 07100 Sassari, Italy; davide.gentili@aulss2.veneto.it

**Keywords:** vaccination, health worker, flu vaccination, vaccine hesitancy

## Abstract

The aim of this work is to evaluate the attitudes, behaviours, and knowledge of health workers employed at an Italian University Hospital on the topic of vaccinations and in regard to flu vaccination. To this end, the study provided for the articulation of a computerised questionnaire on the digital platform EUSurvey which was administered online via e-mail to a sample of 457 health workers, in the period between November 2018 and March 2019. The data were subjected to descriptive and inferential statistical analysis. In particular, a logistic regression analysis was carried out in order to evaluate the relationship between the variables collected and the dichotomous outcome (vaccinated/unvaccinated subjects in the 2018–2019 season). The results, in line with what has been reported by the literature, highlighted that vaccine hesitancy is prevalent also among health workers. Furthermore, according to our study, only 30.6% of the health care workers had the flu vaccination. The survey points out the need to plan educational and informative interventions aimed at changing the attitudes, behaviours, and knowledge of health workers in the field of flu vaccination, for the purpose of protecting the health of healthcare personnel and their patients.

## 1. Introduction

Influenza syndrome is a significant public health problem and a major source of direct and indirect costs due to the management of cases and complications of the disease [1]. Globally, according to the World Health Organisation (WHO), the flu virus affects between 5% and 15% of the adult population every year (from 350 million to 1 billion people), with a mortality rate ranging from 250,000 to 500,000 deaths [1,2,3]. According to the European Centre for Disease Prevention and Control (ECDC), between 4 and 50 million people in Europe each year experience symptomatic flu with between 15,000 and 70,000 related deaths [4]. Ninety percent of deaths occur in subjects over the age of 65, especially among those with underlying chronic clinical conditions [5,6]. In Italy alone, 5–8 million people are affected by flu syndrome each year, the impact of which, in terms of lethality, translates to an estimated 8000 deaths per year [7,8].

In public health, the prevention of seasonal flu is of utmost importance in order to reduce its epidemiological, clinical, and economic impact [9,10]. To date, vaccination is the most effective preventive strategy available for this purpose [6,11]. However, despite this important opportunity for prevention, the percentage of vaccinated individuals is constantly decreasing [2,12].

For the 2018/2019 season, the coverage values recorded in Italy show a percentage of 15.8%, slightly higher than that of the previous season (15.3% in the two-year period 2017–2018). In Sardinia, influenza vaccination coverage per 100 inhabitants is 14.2% [8,12].

With regard to the poor compliance with flu vaccination, numerous studies in the literature show that seasonal flu in a common sense represents a health event whose clinical and social impact is largely underestimated [2,3,13,14].

The altered perception of the health risk, in this context, has a significant impact on health decisions made by the population; in fact, a discrepancy between real danger and perceived risk can lead to inappropriate behaviours that do not comply with the recommended public health measures [15]. According to the “outrage” theory coined by the American sociologist Peter Sandman [16], in the perception of risk, a key role is played by the emotional component surrounding the event.

Influenza is often perceived by the population as an exacerbated or an inapparent risk, compared to the real incidence of the disease [17,18,19,20]. What is more, several studies have shown that an inaccurate perception of the magnitude of an event is based on poor and ineffective risk communication [20,21,22,23,24].

The rampant phenomenon of vaccine hesitancy [25,26,27,28] highlights the need to focus attention on the complexity of the communication processes, which are indispensable for adequate adherence to vaccination. In fact, despite the various initiatives promoted at a national and international level, the spread of distorted information in the media and in particular on the various social networking sites has led to a decrease in vaccination coverage among healthcare workers also [28,29,30,31,32].

Healthcare workers are particularly exposed to the risk of contracting the flu (in a clinical and sub-clinical form) and also transmitting the infection to patients whose underlying conditions increase the risk of complications [2,33,34,35,36]. In the global healthcare landscape, in order to guarantee the protection of patients and the health workers themselves, the Centres of Disease Control and Prevention (CDC), the Advisory Committee on Immunization Practices (ACIP), and the Healthcare Infection Control Practices Advisory Committee (HICPAC) annually recommend anti-influenza vaccination to all healthcare professionals [35,36,37]. In the United States, to optimise the control of influenza in the healthcare environment, the “Healthy People 2020” plan was released, establishing a minimum target of 90% flu vaccination coverage among health workers by 2020 [38].

In Europe, all EU member states have adhered to a minimum target of 75% and the Italian Ministry of Health, in line with what has been established in Europe, has set a minimum threshold of 75% coverage (given the optimal value of 95%) [39,40]; to this end, vaccination is offered actively and free-of-charge to health workers, as well as to all individuals aged sixty-five and over, as well as to the at-risk categories provided for by the 2017–2019 National Vaccination Prevention Plan (Piano Nazionale di Prevenzione Vaccinale) [41]. Nonetheless, coverage for influenza vaccination among healthcare workers falls short of the minimum targets set.

Based on these premises, the present work aims to evaluate the relationship between the knowledge, attitudes, and behaviours of healthcare professionals and their propensity towards flu vaccination, in order to understand which phenomena are most implicated in vaccine hesitancy and to develop targeted strategies to increase vaccination coverage and improve compliance among health workers. To the best of our knowledge, this is the first study to focus on the flu vaccination attitude, behaviours, and knowledge among healthcare workers in Sardinia, Italy.

## 2. Materials and Methods

### 2.1. Study Setting

The present study did not require ethical approval for its observational design according to the Italian law (Gazzetta Ufficiale no. 76 dated 31.3.2008). 

The fact-finding survey was directed towards employees of the University Hospital of Sassari (AOU-SS). The AOU-SS is the main hospital in Sardinia for the number and heterogeneity of its professional resources (2710 employees as of 01.12.2018) and its technological resources and carries out multi-specialist activities of care, teaching, and research for the entire northern territory of Sardinia.

The organisational structure of the hospital is set out in the corporate deed (art. 3 paragraph 1 bis of Legislative Decree no. 502/92 and subsequent amendments), which identifies a total of 77 operational units. Based on the type of activity carried out, these units are grouped into macro-areas: 29 medical areas; 18 surgical areas; 30 services/other [42].

### 2.2. Survey Method

An anonymous self-administered questionnaire was developed on the EUSurvey digital platform (an open-source tool of the European Commission for the construction of surveys and consultations). The questionnaire was tested, adjusted, and validated through a pilot study, carried out on a convenience sample of 40 experts in public health. The internal consistency was assessed with Cronbach’s alpha test. 

To assess the attitudes, behaviours, and knowledge of the AOU-SS staff regarding flu vaccination, the questionnaire was divided into 26 close-ended mandatory questions divided into 4 areas of investigation: 6 personal data questions aimed at classifying the professional profile of the participants; 7 questions related to attitudes; 3 questions about behaviours, and 10 questions about knowledge. For questionnaire compilation, it was compulsory to answer each question.

The questionnaire was administered by sending a URL code via e-mail to AOU-SS employees in the period between November 2018 and March 2019. The general information of the respondents is shown in Table 1. The attitudes, behaviours, and knowledge questions are shown in Table 2 (Results Section). In particular, the questions and the close-ended answers (coded as qualitative data) are reported in the first and second columns of the table, respectively.

### 2.3. Statistical Analysis

Data were entered on Excel (Microsoft Office, Microsoft Corporation, Redmond, WA, USA) and analysed using the STATA software 11 (StatCorp., Austin, TX, USA) and MedCalc (MedCalc Software Ltd., Ostend, Belgium). Qualitative variables were summarised with absolute and relative (percentage) frequencies. 

Logistic regression analyses were performed to assess the relationship between the outcome (vaccination carried out in 2018–2019) and variables related to health workers’ attitudes, behaviours, and knowledge. The outcome was established by attributing a value of 1 if the participant underwent vaccination in 2018–2019, and a value of 0 otherwise. Only the variables that were significant in the first phase of univariate analysis were included in the multivariate model.

A two-tailed *p*-value of less than 0.05 was considered statistically significant.

## 3. Results

The invitation to participate in the compilation of the questionnaire was sent via e-mail to 2270 (83.7%) AOU-SS health workers. There were 457 (20.1%) questionnaires completed and returned.

Cronbach’s alpha reliability test showed a global value of 0.8592, which highlights the good internal consistency of the questionnaire.

The results of the descriptive analysis are shown in Table 2.

### 3.1. Attitudes

Of those interviewed, 55.1% believed that flu vaccination for health workers was a right and a responsibility in the protection of health. In addition, almost all respondents believed that vaccines were a pivotal tool for prevention and were capable of eliminating serious communicable diseases (99.6% and 98.2%, respectively). Moreover, the same interviewees considered anti-influenza vaccination for health workers as protection for patients (93.7%), and most (65.9%) showed no concern about the possible side effects of vaccination. As such, 58.4% of respondents were in favour of mandatory flu vaccination for health workers, while the majority of those not in favour (111 out of 190 respondents) believed that obligation affected individual freedom of choice. Finally, giving greater visibility to the vaccination campaign, together with a specific training activity on the topic of influenza, were considered the best strategies for proposing the flu vaccination to healthcare professionals by almost all interviewees (95.6% and 96.7%, respectively).

### 3.2. Behaviours

As regards the behaviour of the sample interviewed about flu vaccination, the highest percentage of vaccinated subjects (30.6%) regarding the five years observed, was attributable to the 2018/2019 season. Moreover, 44.2% of those unvaccinated in 2018/2019, stated they wished to be vaccinated. What is more, almost half (48.3%) of the respondents stated they had been vaccinated in the past 5 years. At the same time, 45.5% stated they had contracted flu during the past 2 years; of these only 42.3% of cases were absent from work until they had made a complete recovery.

### 3.3. Knowledge

Knowledge among those interviewed concerning flu vaccination showed that, albeit in a low percentage (26.0%), the ineffectiveness in the prevention of seasonal flu was the main reason for failing to adhere to vaccination. Only 52.1% of the respondents were aware of having a greater risk of contracting the disease than the general public, while almost all were aware that there are some at-risk categories, such as those aged over 65 (99.6%) and people with dysmetabolic diseases and diabetes (87.7%). Only 27.1% were aware of the actual incubation period of the disease, just as only 29.1% were aware that the incidence of influenza is higher among subjects <15 years old. By contrast, almost all respondents (93.9%) knew that pneumonia was the most frequent complication of influenza, and more than half (56.9%) knew that the vaccines currently in use protect against type A and type B viruses. 

### 3.4. Logistic Regression Analysis

As regards the univariate and multivariate analysis, the variables relating to questions number 14 and 15 were excluded from the analysis, as all the respondents who were vaccinated in 2018–2019 (outcome) had received flu vaccination also in the last 5 years (at least once). Thus, this led to the exclusion from the model of the variables associated with the outcome due to the obvious collinearity. In the models, the questions relating to knowledge were categorised based on the correctness or otherwise of the answer given (correct answer = 1, otherwise = 0). The questions with nested answers were excluded from the inferential analysis, as they refer to a limited number of respondents and, therefore, are not representative of the sample as a whole. The results are shown in Table 3.

No significant differences were observed for age, gender, and seniority with regard to the knowledge level of the respondents.

**Figure 1:**
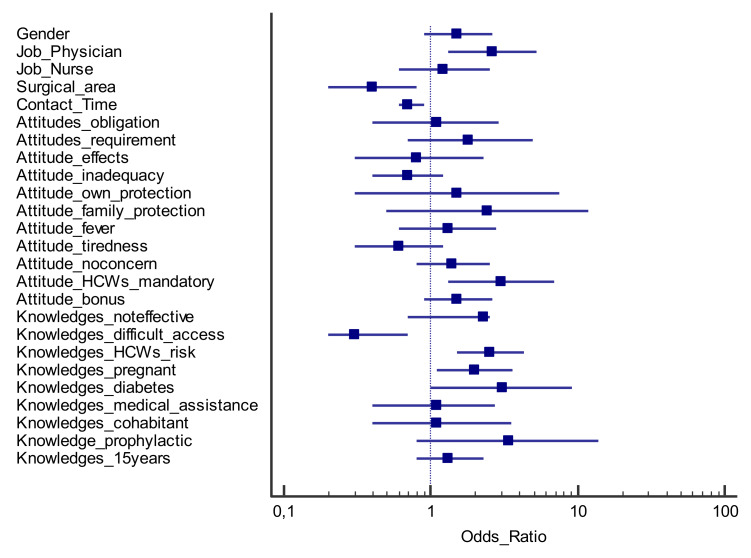
Forest plot reporting the multivariate logistic analysis results; odds ratios and 95% confidence intervals (question_answer) concerning the observed outcome (vaccinated/unvaccinated subjects in the 2018–2019 season).

The variables that were statistically significant from the univariate analysis in relation to the outcome (staff vaccinated in the 2018–2019 season) were selected and included in a multivariate logistic regression model (Figure 1).

Following multivariate inferential analysis, some variables were found to be associated with the outcome. In particular, AOU-SS health workers showed a greater propensity to flu vaccination if: (i) doctors (odds ratio, OR (confidence interval, CI 95%) = 2.6 (1.3–5.2); *p* = 0.007); (ii) in favour of mandatory flu vaccination for their professional category (OR (CI 95%) = 3.0 (1.3–6.9); *p* = 0.011; (iii) aware of their greater risk of developing the disease than that of the general population (OR (CI 95%) = 2.5 (1.5–4.3); *p* = 0.001); (iv) are aware that some categories of people (pregnant women, OR (CI 95%) 2.0 (1.1–3.6); *p* = 0.018, subjects with diabetes or dysmetabolic diseases, OR (CI 95%) = 3.1 (1.0–9.2); *p* = 0.043) are at-risk categories. On the other hand, the health workers who did not get flu vaccination in the 2018–2019 season were those who: i) belonged to the surgical area (OR (CI 95%) = 0.4 (0.2–0.8); *p* = 0.010); ii) spent more time in contact with patients (OR (CI 95%) = 0.7 (0.6–0.9); *p* = 0.005); iii) declared difficulties in accessing vaccination (OR (CI 95%) = 0.3 (0.2–0.7); *p* = 0.002).

## 4. Discussion

The present study presents weaknesses and strengths that are discussed below. In particular, although the survey provided for the administration of a questionnaire to 2270 AOU-SS employees, only 457 questionnaires were returned (20.1%). Therefore, this could underlie a selection bias, because those who are most attentive to vaccination also are those who most likely respond to an interview on it. However, the sample that took part in the survey was mainly made up of healthcare workers who are in direct contact with patients (68.5% of the interviewees spent over 50% of their time in contact with the patient) and that, even if counterintuitive, according to the results from the logistic regression, should be those that less frequently get the flu vaccination. Therefore, these two phenomena, the former in favour and the latter against the vaccination compliance, tend to compensate each other. As a result, 30.6% of respondents stated they had been vaccinated in the current season (2018–2019). This percentage was the highest of the years observed and, although there are no official statistics on the matter, it was greater than the vaccination coverage reported by the most recent observations at national and European level [43,44,45,46]. Furthermore, adding the number of respondents vaccinated in 2018–2019 to the number who expressed willingness to be vaccinated, the forecast of vaccination coverage of the sample observed rose to 60.2%. This data suggests that the health workers who took part in the survey were highly inclined towards flu vaccination. 

Despite potential selection bias, the results that emerged from the inferential analysis are in line with what has been observed in the literature by numerous other authors. For example, as has been found in recent observations, our results showed a significantly higher adhesion to vaccination by medical personnel [46,47,48,49,50,51]. On the contrary, staff working in the surgical area, as well as health workers who spent more time in contact with the patients, showed a lower adhesion to vaccination, which was also statistically significant. This result, found in other observations [52,53,54], is key to the implementation of targeted corrective measures.

As regards the results of the inferential analysis relating to the attitudes of the interviewees, our study, as mentioned, is in line with what has been observed by other authors internationally [47]. In particular, those who declared that they were vaccinated in 2018–2019, were in favour of mandatory influenza vaccination for healthcare professionals. It is interesting to note that most of those who were not in favour of mandatory vaccination believed that this obligation affected individual freedom of choice. Freedom of choice represents one of the main known determinants of vaccine hesitancy among health workers [27,30].

The survey also significantly revealed that those who adhered to vaccination were aware of a greater risk of their developing the disease than the general public, and knew the higher risks of certain categories of people (subjects with dysmetabolic diseases or diabetes; pregnant women). It was in line with what has already been observed by other scholars [55]. 

A noteworthy result regarded the significant difficulty declared by the health workers for easy access to the vaccination service. This aspect is crucial for the development of intervention strategies, which must facilitate access to the service in order to increase coverage [56].

There is also the potential for social desirability bias, as the study participants may have overstated that they are prone to get the flu vaccine to appear more health conscious. However, “threat of disclosure”, pertaining to respondents’ concerns about possible risks, costs, or negative consequences of truthfully reporting a sensitive behaviour was limited by the anonymity and confidentiality of the digital survey administered, thus decreasing the respondent’s concerns in admitting to some taboo (e.g., via confidentiality assurances or clever wording and framing of the sensitive item) [57,58,59].

## 5. Conclusions

In the context of social care, health workers are not exempt from the phenomenon of vaccine hesitancy, which is widespread also in the hospital setting. Immunising health professionals means, on the one hand, protecting the workers and the patients in their care, and on the other, curtailing the spread of infections, while maintaining a high quality of health and care services during epidemics.

Despite being offered actively and free of charge, the anti-influenza vaccination coverage of healthcare professionals continues to remain far below the minimum targets set. To counter this phenomenon, it will be essential to promote communication/information strategies tailored to healthcare professionals. These must be contextually supported by the provision of vaccination directly in the ward in order to overcome the difficulties of access to the service manifested by the respondents. The scheduling of interventions to promote influenza vaccination aimed at susceptible cohorts (e.g., surgical staff and non-medical staff) would enable an increase in vaccination coverage. Indeed, this affirmation is confirmed by the results that emerged regarding the knowledge of the staff interviewed. The results obtained, reveal that opinion tends to be favourable towards vaccination, with clear margins for improvement in terms of attitudes, knowledge, and behaviours and, thus, vaccine compliance.

Furthermore, as the occupational physicians (Ophs) are fundamental to inform the workers about the pros and cons of recommended vaccinations, an interaction with an Oph is also useful to address personal misconceptions and target false beliefs, ultimately increasing the awareness of the potential of the flu vaccine. For this reason, during the last two years, we have worked closely with Ophs to improve adherence to flu vaccination, focusing mainly on improving information (with many field events supported by the vaccinarsinsardegna.org team and also involving the basketball team Dinamo Sassari as a testimonial) and expanding the vaccine distribution network (vaccination in the hospital wards) [60,61]. 

To conclude, although the vaccination coverage targets set at the national level are still distant, the present work highlights which strategies should be implemented to aim for the minimum values foreseen in the near future. The low questionnaire response rate suggested that who more likely responded to the questionnaire were in favour of flu vaccination. Consequently, the vaccination propensity among the healthcare workers we observed might be even lower than expected. 

On the other hand, the actual Covid-19 pandemic could represent an opportunity of fighting the vaccine hesitancy propensity among Italian healthcare workers and AOU-SS healthcare professionals heavily hit by the virus.

## Figures and Tables

**Table 1:** Participants’ general information.

General Information	Answers (Yes/No)	Yes (%)
1. Gender	Male	168/457 (36.8)
2. Age (years)	25–34	37/457 (8.1)
	35–44	102/457 (22.3)
	45–54	167/457 (36.5)
	55–64	143/457 (31.3)
	>65	8/457 (1.8)
3. Job	Physician	163/457 (35.7)
	Nurse	178/457 (38.9)
	Other	116/457 (25.4)
4. Area	Medical	151/457 (33.0)
	Surgical	106/457 (23.2)
	Service/other	200/457 (43.8)
5. Seniority (years of service)	0–9	110/457 (24.1)
	10–19	129/457 (28.2)
	20–29	156/457 (34.1)
	>30	62/457 (13.6)
6. Contact time (% working time spent in contact with the patient)	0–25	95/457 (20.8)
	26–50	49/457 (10.7)
	51–75	90/457 (19.7)
	76–100	223/457 (48.8)

**Table 2:** Descriptive analysis of the variables inherent to the health workers’ general information, attitudes, behaviours, and knowledge. The first column on the left shows the questions of the questionnaire, while the possible answers (yes/no) are shown in the “answers” column.

**Attitudes**	**Answers (Yes/No)**	**Nested Answers (%)**	**Yes (%)**
7. Do you believe that vaccinations for healthcare professionals constitute:	an obligation from a professional responsibility viewpoint?	-	188/457 (41.1)
	a right and a responsibility in the defence and protection of health?	-	252/457 (55.1)
	an indispensable requirement for working in the health sector?	-	187/457 (41.0)
8. What are your beliefs about the role of vaccinations in individual and collective human health?	Vaccines are often linked to serious side effects	-	58/457 (12.7)
	The effects of vaccination are unknown	-	46/457 (10.1)
	The potential risks outweigh the potential benefits	-	67/457 (14.7)
	Vaccines represent a legacy of achievement in the prevention of infectious diseases	-	455/457 (99.6)
	Vaccines are important for reducing or eliminating serious infectious diseases	-	449/457 (98.2)
	It is better to “go through” the disease rather than to vaccinate against it	-	13/457 (2.8)
9. Which of the following factors do you consider to be determinants of vaccination refusal/uncertainty for healthcare professionals?	Mistrust of vaccines and vaccination practice	-	278/457 (60.8)
	Scepticism, negative perception of the importance of vaccines	-	314/457 (68.7)
	Inadequacy of the practice for the populations’ needs	-	185/457 (40.5)
	Contextual influences of a social, cultural, economic, and political nature	-	354/457 (77.5)
	Other types of influences (time, cost, and lack of information)	-	351/457 (76.8)
10. Do you believe flu vaccination for healthcare professionals is:	protection for yourself?	-	278/457 (60.8)
	protection for your family members?	-	407/457 (89.1)
	protection for patients?	-	428/457 (93.7)
11. Which of the following (common, rare, or only theoretical) side effects to some extent related to flu vaccination worry you?	Pain at the injection site	-	55/457 (12.0)
	Fever	-	97/457 (21.2)
	Feeling of tiredness and/or fatigue	-	119/457 (26.0)
	Diseases of the peripheral nervous system or Guillain–Barré syndrome	-	109/457 (23.9)
	Allergic manifestations	-	134/457 (29.3)
	No concern, the reactions are transient, minor, and very rare	-	301/457 (65.9)
12. Would you be in favour of mandatory flu vaccination for health workers as a fundamental requirement for working within the national health system?	-	-	267/457 (58.4)
* 12.a Why would you be against it?	Obligation affects individual freedom of choice	111/190 (58.4)	-
	Obligation is limited to specific professional categories	50/190 (26.3)	-
	Obligation would expose me to a risk I had not chosen to take	29/190 (15.3)	-
13. In a hospital setting, what do you think could be the best strategy to propose flu vaccination to health professionals?	Make vaccination mandatory	-	244/457 (53.4)
	Give greater visibility to the vaccination campaign	-	437/457 (95.6)
	Award a bonus to employees who decide to get vaccinated	-	149/457 (32.6)
	Give specific training on the topic of influenza	-	452/457 (96.7)
**Behaviours**	**Answers (Yes/No)**	**Nested Answers (%)**	**Yes (%)**
14. Did you get flu vaccination during these periods?	2014/2015	-	119/457 (26.0)
	2015/2016	-	104/457 (22.8)
	2016/2017	-	108/457 (23.6)
	2017/2018	-	132/457 (28.9)
	2018/2019	-	140/457 (30.6)
** 14.a Are you planning to be vaccinated against flu in 2018–2019?	-	140/317 (44.2)	-
15. Did you get flu vaccination during the last 5 years?			221/457 (48.3)
16. Did you contract flu in the last 2 years?	-	-	208/457 (45.5)
*** 16.a If you have contracted flu in the last 2 years, what action did you take?	Absence from work until complete recovery	88/208 (42.3)	-
**Knowledge**	**Answers (Yes/No)**	**Nested Answers (%)**	**Yes (%)**
17. Which of the following answers is a reason for not adhering to flu vaccination?	The flu vaccine is not entirely safe for health	-	45/457 (9.8)
	The flu vaccine is not effective in preventing seasonal flu		119/457 (26.0)
	The flu vaccine can cause serious side effects		51/457 (11.2)
	Difficulty accessing flu vaccination		82/457 (17.9)
	Cost of the vaccine		31/457 (6.8)
18. Do you believe that given your professional activity, the risk of contracting the flu compared to the general public is:	Greater than the general public	-	238/457 (52.1)
	Less than or equal to the general public		219/457 (47.9)
19. The sources of influenza infection are:	Healthy carriers	-	229/457 (50.1)
	Chronic carriers	-	149/457 (32.6)
	Asymptomatic carriers	-	284/457 (62.1)
	Subjects with no other clinical symptoms	-	130/457 (28.4)
20. Which flu vaccines are currently in use in Italy?	Attenuated	-	265/457 (57.9)
	Split		244/457 (53.3)
	Inactivated		300/457 (65.6)
	Subunit		208/457 (45.5)
	Adjuvanted		254/457 (55.5)
21. Flu vaccination is recommended in the following risk categories:	over 65s	-	455/457 (99.6)
	pregnant women	-	310/457 (67.8)
	subjects with diseases of the haematopoietic organs or chronic circulatory, respiratory, or renal conditions	-	439/457 (96.1)
	subjects with diabetes or other dysmetabolic diseases	-	401/457 (87.7)
	subjects with congenital or acquired illnesses that compromise the immune system	-	378/457 (82.7)
	subjects who require frequent medical assistance	-	400/457 (87.5)
	cohabitants of at-risk subjects	-	418/457 (91.5)
22. Which of these measures are recommended in primary flu prevention?	Standard immunoglobulins	-	80/457 (17.5)
	Specific immunoglobulins	-	101/457 (22.1)
	Prophylactic vaccination	-	431/457 (94.3)
	Hand washing	-	437/457 (95.6)
	Use of medical masks by flu patients	-	388/457 (84.9)
23. The incubation period of influenza is:	1 week	-	124/457 (27.1)
	6–12 h		37/457 (8.1)
	1–2 days		282/457 (61.7)
	2 weeks		14/457 (3.1)
24. Influenza has a higher incidence in those aged:	<15 years old	-	133/457 (29.1)
	15–64 years old		80/457 (17.5)
	over 64 years old		244/457 (53.4)
25. What is the most frequent complication of flu?	Pneumonia	-	429/457 (93.9)
	Myocarditis/pericarditis		10/457 (2.2)
	Myositis		5/457 (1.1)
	Reye syndrome		3/457 (0.6)
	Encephalitis		4/457 (0.8)
	Death		3/457 (0.7)
	I do not know		3/457 (0.7)
26. The influenza vaccines in use protect against viruses of type:	A and B	-	260/457 (56.9)
	only A		49/457 (10.7)
	A, B, and C		148/457 (32.4)

* Question 12.a was made accessible only to those who gave a negative answer to question no. 12, thus excluding those who gave affirmative answers (nested answers). ** Question 14.a was made accessible only to those who gave a negative answer to question no. 14, thus excluding those who gave affirmative answers (nested answers). *** Question 16.a was made accessible only to those who gave a negative answer to question no. 16, thus excluding those who gave affirmative answers (nested answers).

**Table 3:** Univariate and multivariate analysis evaluating the relationships between the independent variables and the outcome vaccinated/unvaccinated subjects in the 2018–2019 season.

Questionnaire Area (Item No.)	Answers	Univariate Analysis		Multivariate Analysis	
**General Information**	**(Yes/No)**	**Odds Ratio (95% CI)**	***p*** **-Value**	**Odds Ratio (95% CI)**	***p*** **-Value**
1. Gender	Male	2.1 (1.4–3.2)	0.000	1.5 (0.9–2.6)	0.108
2. Age (years)	25–34	1.2 (1.0–1.5)	0.071	-	-
	35–44				
	45–54				
	55–64				
	>65				
3. Job	Physician	2.6 (1.7–4.0)	0.000	2.6 (1.3–5.2)	0.007
	Nurse	0.4 (0.3–0.7)	0.000	1.2 (0.6–2.5)	0.578
	Other	0.8 (0.5–1.2)	0.291	-	-
4. Area	Medical	1.3 (0.9–2.0)	0.216	-	-
	Surgical	0.4 (0.2–0.7)	0.002	0.4 (0.2–0.8)	0.010
	Service/other	1.4 (0.9–2.1)	0.114	-	-
5. Seniority (years of service)	0–9	1.1 (0.9–1.4)	0.187	-	-
	10–19				
	20–29				
	>30				
6. Contact time (% working time spent in contact with the patient)	0–25	0.8 (0.7–0.9)	0.009	0.7 (0.6–0.9)	0.005
	26–50				
	51–75				
	76–100				
**Attitudes**	**(Yes/No)**	**OR (95% CI)**	***p*** **-Value**	**OR (95% CI)**	***p*** **-Value**
7. Do you believe that vaccinations for healthcare professionals constitute:	an obligation from a professional responsibility viewpoint?	2.9 (1.4–5.8)	0.004	1.1 (0.4–2.9)	0.790
	a right and a responsibility in the defence and protection of health?	3.9 (0.9–17.2)	0.071	-	-
	an indispensable requirement for working in the health sector?	4.4 (2.1–9.1)	0.000	1.8 (0.7–4.9)	0.244
8. What are your beliefs about the role of vaccinations in individual and collective human health?	Vaccines are often linked to serious side effects	0.6 (0.3–1.2)	0.149	-	-
	The effects of vaccination are unknown	0.4 (0.2–0.9)	0.021	0.8 (0.3–2.3)	0.739
	The potential risks outweigh the potential benefits	0.7 (0.4–1.2)	0.196	-	-
	Vaccines represent a legacy of achievement in the prevention of infectious diseases	Omitted due to collinearity	-	-	-
	Vaccines are important for reducing or eliminating serious infectious diseases	3.1 (0.4–25.8)	0.287	-	-
	It is better to “go through” the disease rather than to vaccinate against it	0.7 (0.2–2.5)	0.551	-	-
9. Which of the following factors do you consider to be determinants of vaccination refusal/uncertainty for healthcare professionals?	Mistrust of vaccines and vaccination practice	1.1 (0.7–1.6)	0.703	-	-
	Scepticism, negative perception of the importance of vaccines	1.2 (0.8–1.9)	0.405	-	-
	Inadequacy of the practice with respect to the populations’ needs	0.6 (0.4–0.9)	0.016	0.7 (0.4–1.2)	0.184
	Contextual influences of a social, cultural, economic, and political nature	1.4 (0.9–2.3)	0.179	-	-
	Other types of influences (time, cost, and lack of information)	1.1 (0.7–1.8)	0.723	-	-
10. Do you believe flu vaccination for healthcare professionals is:	protection for yourself?	5.5 (1.9–15.6)	0.001	1.5 (0.3–7.5)	0.639
	protection for your family members?	7.9 (2.4–26.0)	0.001	2.4 (0.5–11.7)	0.266
	protection for patients?	Omitted due collinearity	-	-	-
11. Which of the following (common, rare, or only theoretical) side effects to some extent related to flu vaccination worry you?	Pain at the injection site	1.0 (0.6–19)	0.962	-	-
	Fever	0.6 (0.3–0.9)	0.032	1.3 (0.6–2.8)	0.478
	Feeling of tiredness and/or fatigue	0.4 (0.3–0.7)	0.001	0.6 (0.3–1.2)	0.138
	Diseases of the peripheral nervous system or Guillain–Barré syndrome	1.0 (0.7–1.6)	0.885	-	-
	Allergic manifestations	0.8 (0.5–1.2)	0.261	-	-
	No concern, the reactions are transient, minor, and very rare	1.8 (1.1–2.7)	0.012	1.4 (0.8–2.5)	0.202
12. Would you be in favour of mandatory flu vaccination for health workers as a fundamental requirement for working within the national health system?	-	4.4 (2.8–7.1)	0.000	3.0 (1.3–6.9)	0.011
13. In a hospital setting, what do you think could be the best strategy to propose flu vaccination to health professionals?	Make vaccination mandatory	3.5 (2.2–5.4)	0.000	0.8 (0.4–1.8)	0.585
	Give greater visibility to the vaccination campaign	4.2 (0.9–18.2)	0.058	-	-
	Award a bonus to employees who decide to get vaccinated	1.7 (1.1–2.5)	0.014	1.5 (0.9–2.6)	0.114
	Give specific training on the topic of influenza	1.8 (0.5–6.5)	0.370	-	-
**Behaviours**	**(Yes/No)**	**OR (95% CI)**	***p*** **-Value**	**OR (95% CI)**	***p*** **-Value**
16. Did you get influenza in the last 2 years?	-	1.1 (0.7–1.6)	0.794	-	-
**Knowledge**	**(Yes/No)**	**OR (95% CI)**	***p*** **-Value**	**OR (95% CI)**	***p*** **-Value**
17. Which of the following answers is a reason for not adhering to flu vaccination?	The flu vaccine is not entirely safe for health	1.4 (0.8–2.7)	0.275	-	-
	The flu vaccine is not effective in preventing seasonal flu	0.5 (0.3–0.8)	0.002	1.3 (0.7–2.5)	0.436
	The flu vaccine can cause serious side effects	1.8 (0.6–1.9)	0.903	-	-
	Difficulty accessing flu vaccination	0.5 (0.3–0.9)	0.034	0.3 (0.2–0.7)	0.002
	Cost of the vaccine	0.6 (0.3–1.5)	0.317	-	-
18. Do you believe that given your professional activity, the risk of contracting the flu compared to the general public is:	Greater	3.1 (2.1–4.8)	0.000	2.5 (1.5–4.3)	0.001
19. The sources of influenza infection are:	Healthy carriers	1.2 (0.8–1.7)	0.435	-	-
	Chronic carriers	0.7 (0.4–1.1)	0.099	-	-
	Asymptomatic carriers	1.2 (0.8–1.9)	0.296	-	-
	Subjects with no other clinical symptoms	1.2 (0.8–1.7)	0.462	-	-
20. Which flu vaccines are currently in use in Italy?	Attenuated	1.4 (0.9–2.0)	0.140	-	-
21. Flu vaccination is recommended in the following risk categories:	Over 65s	Omitted due collinearity	-	-	-
	Pregnant women	2.7 (1.7–4.4)	0.000	2.0 (1.1–3.6)	0.018
	subjects with diseases of the haematopoietic organs or chronic circulatory, respiratory, or renal conditions	3.7(0.8–16.2)	0.086	-	-
	subjects with diabetes or other dysmetabolic diseases	5.2 (2.0–13.3)	0.001	3.1 (1.0–9.2)	0.043
	subjects with a congenital or acquired illness which compromise the immune system	1.3 (0.7–2.2)	0.391	-	-
	subjects who require frequent medical assistance	2.6 (1.2–5.5)	0.012	1.1 (0.4–2.7)	0.918
	cohabitants of at-risk subjects	2.6 (1.1–6.3)	0.037	1.1 (0.4–3.5)	0.826
22. Which of these measures are recommended in primary flu prevention?	Standard immunoglobulins	1.6 (0.9–2.9)	0.084	-	-
	Specific immunoglobulins	1.7 (0.9–2.8)	0.054	-	-
	Prophylactic vaccination	3.6 (1.1–12.1)	0.041	3.4 (0.8–13.9)	0.089
	Hand washing	1.3 (0.5–3.8)	0.577	-	-
	Use of medical masks by flu patients	0.7 (0.4–1.2)	0.170	-	-
23. The incubation period of influenza is:	1 week	1.4 (0.9–2.2)	0.110	-	-
24. Influenza has a higher incidence in those aged:	<15 years old	1.6 (1.0–2.4)	0.039	1.3 (0.8–2.3)	0.303
25. What is the most frequent complication of flu?	Pneumonia	1.1 (0.5–2.7)	0.807	-	-
26. The influenza vaccines in use protect against viruses of type:	A and B	1.4 (0.9–2.1)	0.088	-	-
